# A cohort study investigating differences in skeletal muscle mitochondrial function between prostate cancer patients and healthy controls

**DOI:** 10.1186/s12885-026-16164-2

**Published:** 2026-05-21

**Authors:** Thomas FF Smart, Joseph J Bass, Hannah Crossland, Jake Cox, Philip J Atherton, Jon N Lund, Bethan E Phillips

**Affiliations:** 1 Centre of Metabolism, Ageing and Physiology (COMAP), MRC-Versus Arthritis Centre for Musculoskeletal Ageing Research, School of Medicine, University of Nottingham Medical School at Derby, Derby, DE22 3DT United Kingdom; 2 Department of Surgery, Royal Derby Hospital, Derby, United Kingdom

**Keywords:** Cancer, Mitochondria, Skeletal muscle, Prostate

## Abstract

**Background:**

There has been an increasing prevalence of prostate cancer worldwide, and therefore a growing clinical need to understand and maintain whole body health in this cohort. There is currently limited evidence of the underlying physiology which leads to certain patients developing cancer cachexia. Based on pre-clinical evidence, skeletal muscle (SKM) mitochondria are suspected to play an integral role, however to date, there is little human data to support this. This study compares SKM mitochondrial oxidative phosphorylation (OXPHOS) in prostate cancer patients due to undergo treatment with curative intent to healthy volunteers, with a hypothesis that this would be lower in the cancer cohort.

**Methods:**

Twelve prostate cancer patients and 8 disease-free males matched for age and body mass index (BMI) were recruited to this study. All participants completed assessments of physical function using clinically recognised tools (handgrip strength, timed up-and-go, and short physical performace battery) and muscle architecture via ultrasound. All participants also had a SKM (vastus lateralis) biopsy collected. Using a high-resolution respirometer (OROBOROS) and standardised substrate uncoupled inhibitor titration (SUIT) protocol, SKM OXPHOS was assessed, with the biopsy also analysed for citrate synthase activity and gene expression via RT-PCR.

**Results:**

Despite our groups being well-matched in terms of age and BMI, and there being no differences in SKM physical function or architecture, extrinsic OXPHOS was significantly lower in the prostate cancer cohort compared to healthy controls (maximal complex I activity: 47.1 ± 19.7 vs. 27.1 ± 12.1, *p* = 0.01; maximal complex I + II activity: 74.8 ± 26.7 vs. 50.7 ± 13.4, *p* = 0.01; maximal activity: healthy: 95.9 ± 37.4 vs. 62.1 ± 16.3, *p* = 0.02). The same was true for Respiratory Control Ratio (2.7 ± 0.9 vs. 1.8 ± 0.9, *p* = 0.04). There was no difference in intrinsic OXPHOS between the groups.

**Conclusion:**

This study found lower SKM extrinsic OXPHOS in those with prostate cancer, in the absence of muscle mass or functional loss, when compared to age-matched disease-free volunteers. This appears to be due to changes in mitochondrial content or remodelling, with preservation of intrinsic function.

**Supplementary Information:**

The online version contains supplementary material available at 10.1186/s12885-026-16164-2.

## Background

With worldwide life expectancies increasing, there is a growing prevalence of diseases associated with older age. For example, in the UK alone, there are an estimated 363,000 new cancer cases each year [1]. Specific to prostate cancer, the UK has seen a stark rise in prevalence over the last decade resulting in over 55,000 new diagnoses each year. Exemplifying the magnitude of this increased prevalence, the UK National Prostate Cancer Audit State of the Nation Report (2024) highlighted a 25% increase in prostate cancer patients between 2019 and 2023 [2].

Cachexia, defined as unintentional weight loss of > 5%, or > 2% in those with already low body weight [3], is a common condition in those affected by cancer, with ~ 50–70% of those with a cancer diagnosis impacted by this condition [4, 5]. Characterized by a loss of skeletal muscle (SKM) mass which may be accompanied by a loss of fat mass [6], and which cannot be fully explained by anorexia [3], cancer cachexia has significant implications for numerous clinical outcomes. Indeed, increased morbidity, decreased independence, decreased quality of life, and mortality are all associated with cancer cachexia [5, 7, 8]. Cancer cachexia also implicates upon chemotherapy treatment with increased likelihood of side effects, decreased cycles completed, and decreased survival rates [9, 10]. Finally, in relation to surgical outcomes, cancer cachexia leads to increased length of stay, increased morbidity, and increased mortality [11, 12].

Despite growing interest in understanding the mechanisms of cancer cachexia, evidenced by pre-clinical studies [13] and review articles attempting to amalgamate evidence [14], the full underlying pathophysiology of cancer cachexia is not yet understood. In uncomplicated terms, it is accepted to be an energy balance disorder in which a decrease in oral consumption of calories, combined with metabolic disturbance leads to increased energy expenditure, resulting in negative energy and protein balance [15]. The presence of malignancy is estimated to place an extra energy demand on the host of between 100 and 1400 kcal/day depending upon cancer type and load [16], with urological cancers demonstrated to increase resting energy expenditure by ~4 kJ/kg fat free mass/day [17]. Further, the additional demand for glucose associated with malignancy leads to increased hepatic gluconeogenesis resulting in proteolysis of SKM to supply amino acids, thus driving the SKM wasting seen in cachexia [18].

It has been suggested that the increased energy expenditure seen in cancer cachexia is caused by alterations to SKM mitochondria [19], and more specifically mitochondrial oxidative phosphorylation (OXPHOS) [7]. For example, using a mouse model of Lewis lung cancer, Brown et al., demonstrated that SKM mitochondrial dysfunction preceded declines in the cachexia phenotype [20]. Similarly, Hepple and colleagues conducted a study in mice using the C26-induced colon cancer model, in which they demonstrated reduced maximal SKM mitochondrial respiration and reduced mitochondrial coupling in the cancer model, linked to SKM atrophy [21].

To date, there is only a single publication directly exploring the impact of cancer on SKM OXPHOS function in humans [22]. Although this study did report lower SKM OXPHOS in cancer patients, it comprised a series of cases across a broad range of cancer types. In addition, the patients in this study all had metastatic disease and were receiving palliative chemotherapy, rendering it difficult to draw conclusions to the observed mitochondrial alterations (i.e., disease vs. treatment).

Therefore, the aim of this study was to investigate SKM mitochondrial function, gross SKM structure, and physical function in those with non-metastatic prostate cancer scheduled for surgery with curative intent, compared to age-matched disease-free controls. The hypothesis being that skeletal muscle OXPHOS function will be lower in the cancer cohort when compared to healthy volunteers.

## Methods

This study was reviewed and approved by an NHS research ethics committee (IRAS Project ID 275264), with approval for the control group granted by the University of Nottingham Faculty of Medicine and Health Research Ethics Committee (FMHS 407–1910). The study was conducted in accordance with the Declaration of Helsinki and registered at www.clinicaltrials.gov (NCT04558398, NCT05934760).

### Patient identification

Potentially eligible cancer patients were identified at weekly multidisciplinary team meetings within a single NHS trust. Only individuals planned for curative surgery for prostate malignancy, with no neoadjuvant chemotherapy were deemed eligible. After recruitment of the cancer patients was complete, disease-free age and body mass index (BMI)-matched male volunteers were recruited from the local community via posters and community groups.

After provision of a participant information sheet and the opportunity to ask questions, all potential participants underwent a full pre-study screening visit, including measures of height and weight, blood analysis (U&E’s, LFT’s, TFT’s and coagulation profiles), and an ECG to ensure eligibility. Exclusion criteria included current participation in a formal exercise regime, a BMI < 16.5 or > 35 kg/m^2^, active cardiovascular disease (i.e. uncontrolled hypertension, angina, heart failure or recent cardiac event), cerebrovascular disease, respiratory disease (significant COPD or uncontrolled asthma), clotting dysfunction or current use of anticoagulants, and for cancer patients those requiring or receiving neoadjuvant chemotherapy. All potential participants provided written informed consent at this session.

### Study day procedures

After confirmation of eligibility, participants were asked to attend the research facility at 0830 h following an overnight fast (water *ad libitum*). The study day commenced with three low-intensity assessments of physical function: (i) handgrip strength, (ii) timed up-and-go (TUG), (iii) and the short physical performance battery (SPPB), each carried out according to standard protocols [23, 24]. Ultrasound was then performed to assess *m. vastus lateralis* (VL) architecture, including mid-point cross sectional area (CSA), muscle thickness (M_T_), fascicle length (F_L_) and pennation angle (P_A_) [25]. A standardised light breakfast was then provided 60-minutes before a conchotome biopsy of the *m. vastus lateralis* was taken under local anaesthetic as previously described [26]. The muscle biopsy was cleaned in PBS solution prior to half of the muscle (~ 25 mg) being immediately snap frozen in liquid nitrogen for storage at -80 °C prior to real-time polymerase chain reaction (RT-PCR) and citrate synthase (CS) activity analysis. The remaining half was immediately used for SKM OXPHOS analysis.

### Analysis methods

#### Muscle ultrasound

Muscle architecture images were captured using an ultrasound machine (MyLab™50 scanner, Esaote, Italy) with wide (LA923, Esaote) and narrow (LA523, Esaote) linear array probes. Briefly, participants lay supine before the VL was scanned at 50% of its length, determined as the distance between the greater trochanter and mid-point of the patella. The muscle boundaries were determined by ultrasound and muscle architectural images collected at 50% of the width. These anatomical landmarks were chosen to standardise scanning location and take into account variation in leg length between participants. Muscle cross-sectional area (CSA), thickness (M_T_), pennation angle (P_A_) and fascible length (F_L_) were analysed via ImageJ software (NIH, USA) as previously described [25–27].

#### RT-PCR

Total RNA was extracted from the muscle tissue using TRI Reagent^®^ (Sigma-Aldrich) following the manufacturer’s instructions, with RNA purity (A260/A280 and A260/A230 ratios) and concentration assessed using a NanoDrop spectrophotometer (Thermo Fisher Scientific). cDNA was synthesized from the extracted RNA using the High-Capacity cDNA Reverse Transcription Kit (Applied Biosystems™), using 1 µg total RNA. PCR amplification was performed to analyze specific genomic regions of interest. For each reaction, 1 µl of cDNA was used as a template, in a final reaction volume of 7 µl containing 1x PowerUp™ SYBR™ Green Master Mix (Applied Biosystems™) and 0.3 µM of each forward and reverse primer. PCR amplification was carried out using a ViiA™ 7 Real-Time PCR machine, with the following thermal cycling conditions: 50 °C for 2 min, followed by an initial denaturation at 95 °C for 2 min, then 40 cycles of denaturation at 95 °C for 1 s, and annealing/extension at 60 °C for 30 s.

PCR targets included mitochondrial fission (Dynamin-related protein 1 (DNM1L), Fission 1 protein (FIS1)), and fusion targets (Mitofusins (MFN1, MFN2), Optic Atrophy 1 (OPA1)), as well as CS, Cyclooxygenase-2 (COX-II), and PGC-1α (Peroxisome proliferator-activated receptor gamma coactivator 1-alpha) (Supplementary information 1). Gene expression is displayed as the relative change of expression compared to GAPDH (which was validated as being stable in mRNA expression across experimental groups using the 2(-Delta Delta C(T)) method [28].

#### CS activity

For CS activity, muscle samples were mechanically homogenised in ice-cold RIPA buffer (50mM Tris HCl PH 7.4, 150mM NaCl, 1% Triton X-100, 0.5% Sodium deoxylcholate, 0.1% SDS). Samples were centrifuged at 11,000 g for 5 min at 4℃, and the supernatant was removed and quantified using Nanodrop. CS illuminescence assay was performed in 96-well microplate format, incubated at 30 °C, with samples run in duplicate as previously published [29]. In brief, 5 µl of sample was added to a master mix (12.5 µl 1mM TrisHCl, 25 µl 1mM dTNB, 2 µl 12.5mM Acetyl-CoA, 195.5 µl H_2_O) prior to baseline measurement of 412 nm illuminance every 30-seconds for 3-minutes. 5 µl of 5mM oxaloacetate acid was then added, before further 412 nm illuminance measures every 30-seconds for 5-minutes. CS activity was calculated according to rate change of illuminance, enabling estimation of intrinsic mitochondrial OXPHOS function by the normalisation of oxygen flux values to CS activity per weight of tissue [30].

#### High-resolution respirometry

A standardised substrate uncoupled inhibitor titration (SUIT) protocol, designed for use in permeabilized muscle fibres was used [31]. This method allows for the assessment of both coupled OXPHOS and maximal mitochondrial electron flux and establishes optimal tricarboxylic acid cycle (TCA) cycle activity. Rates of oxygen consumption for each sample were measured at 37 °C using an Oxygraph-2k high-resolution respirometer (Oxygraph 2-K, Oroboros Instruments Corp.) with oxygen levels maintained between 250–450µM.

The biopsy tissue was washed in BIOPS buffer (2.77 mM CaK2EGTA, 7.23 mM K2EGTA, 5.77 mM Na2ATP, 6.56 mM MgCl2-6H2O, 20 mM taurine, 15 mM Na2-phosphocreatine, 20 mM imidazole, 0.5 mM dithiothreitol, 50 mM 2-(N-Morpholino)ethanesulfonic acid hydrate, pH 7.1) prior to separation into myofiber bundles and permealisation in BIOPS containing 50µM/mL saponin for 30 min at 4℃. Permeabilised fibres (~ 2–3 mg) were placed in each high-resolution respirometer chamber containing MiR05 medium (Oroboros Instruments) for high-resolution respirometry measurement [31]. Briefly, titration of 5mM pyruvate and 10 mM glutamate allowed measurement of complex I linked leak respiration (Leak), before 5mM ADP for complex I linked oxidative phosphorylative respiration (COM1). Outer mitochondrial membrane integrity was assessed through addition of 10mM cytochrome C, with samples demonstrating a > 10% change in respiration excluded from analysis. 10mM glutamate was added before 10mM succinate to allow Complex I and II linked respiration (COM2) measurement. Maximal electron transfer capacity (MAXIMAL) was determined through serial titration of 1mM FCCP (carbonyl cyanide-p-trifluoromethoxyphenylhydrazone, before addition of 5µM antimycin A to assess non-mitochondrial respiration. Oxygen Flux was measured per milligram of muscle weight (termed extrinsic OXPHOS function), with the aforementioned CS activity used to assess intrinsic mitochondrial OXPHOS function. Samples were run in duplicate, with a minimum of 60% concordance required between chambers. Subsequent analysis allows respiratory control ratio (RCR) to be ascertained via the normalisation of coupled complex I function with baseline LEAK which demonstrates the ability for the mitochondria to switch from resting to active state. Succinate control ratio (SCR) is ascertained by the normalisation of complex I + II to complex I function, representing the relative contribution of complex I + II to the Electron transport chain [32].

### Statistical analysis

Statistical analysis was conducted in Graphpad Prism (GraphPad Version 10.2.0, USA) with all results tested for normality. Where normal distribution was demonstrated an unpaired t-test or one-way ANOVA was performed as appropriate. Where normal distribution was not demonstrated a non-parametric Mann-Whitney test or Kruskal-Wallis test was performed. All results are displayed as mean ± SEM for parametrically distributed data, and median, interquartile range (IQR) when non-parametric. Significance was accepted as *p* < 0.05.

## Results

### Participant demographics

Twelve prostate cancer patients (66 ± 6y, range: 51-74y, 27.2 ± 5.2 kg/m^2^. range: 20.7–34.6 kg/m^2^) and 8 disease-free control participants (69 ± 6y, range: 60–77, 25.9 ± 2.0 kg/m^2^, range: 24.1–30.5 kg/m^2^) were recruited to this study, with no significant difference in age (*p* = 0.33) or BMI (*p* = 0.51) between the groups.

Within the cancer cohort, 9 participants had gleason (3 + 4)7, 2 participants (4 + 3)7, and 1 participant (4 + 4)8; 7 participants had T2c disease, 2 - T3a, 1 - T3b, 1 – T2a, 1- T2x. The most common co-morbidity in both groups was hypertension (3 within cancer cohort, 2 within healthy cohort).

### Physical function

**Fig. 1:**
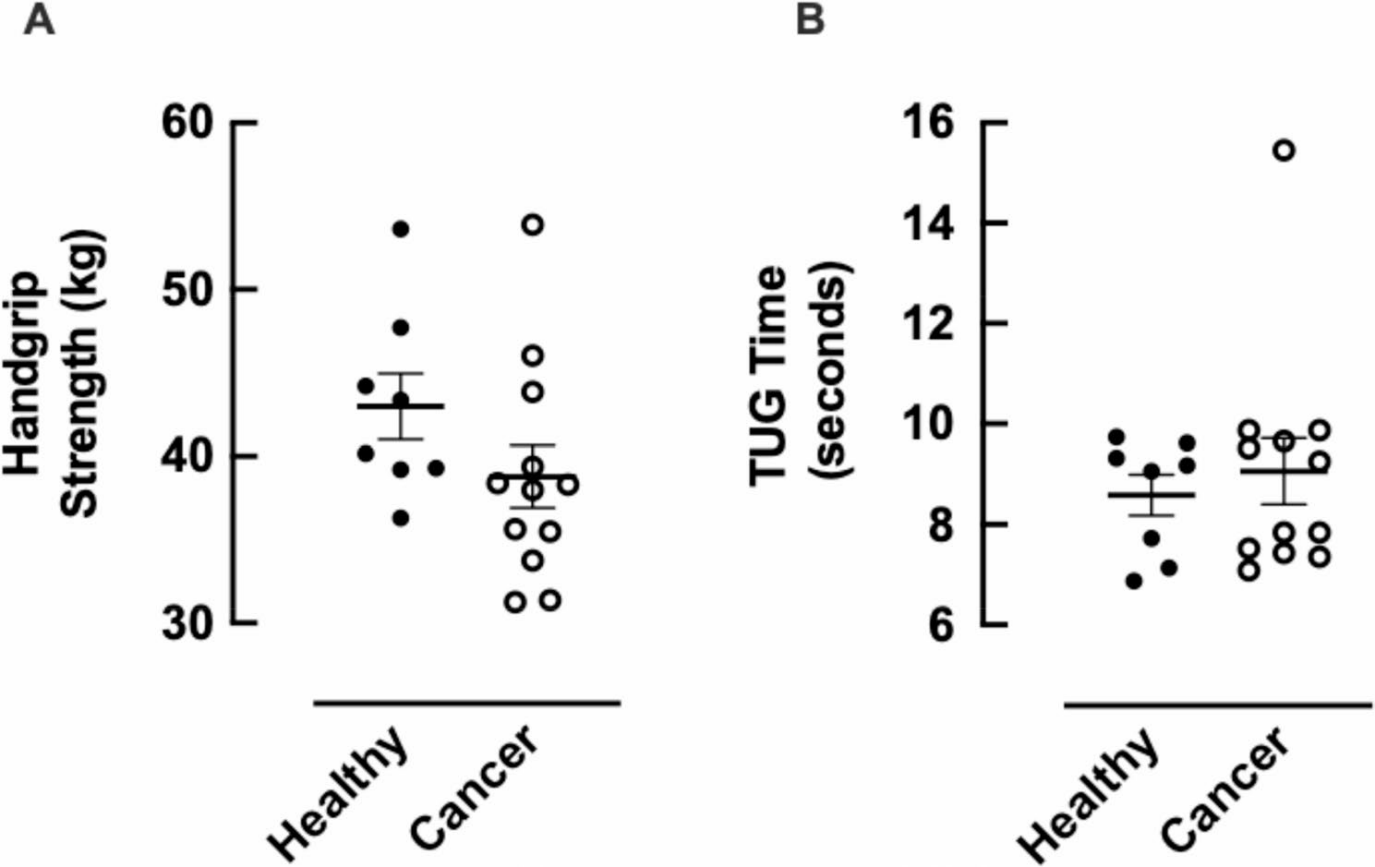
Handgrip strength (**A**) and timed up-and-go (TUG) time (**B**) in prostate cancer patients (*n* = 12) and healthy individuals (*n* = 8) matched for sex (male), age and body mass index. Data are mean ± SEM. Statistical analysis via parametric unpaired t-test

There was no statistically significant difference in handgrip strength (healthy: 43.0 ± 5.6 vs. cancer: 38.8 ± 6.5 kg, *p* = 0.15) (Fig. 1A), TUG (healthy: 8.6 ± 1.2 vs. cancer: 9.1 ± 2.3 s, *p* = 0.59) (Fig. 1B) or SPPB (healthy: 12.0 (IQR 10–12) vs. cancer: 12.0 (IQR 11–12)) between the groups, with no participant in either group classified as frail according to SPPB score.

### Muscle architecture

There was no difference in any aspect of *m. vastus lateralis* muscle architecture between the cancer patients and healthy controls (Table 1).

**Table 1:** Vastus lateralis muscle architecture in prostate cancer patients (*n* = 12) and healthy individuals (*n* = 8) matched for sex (male), age and body mass index

	Healthy controls	Cancer patients	*P*-value
CSA (mm^2^)	20.0 ± 7.0	19.4 ± 4.0	*p*=0.81
M_T_ (cm)	2.4 ± 0.6	2.0 ± 0.5	*p* = 0.17
P_A_ (degrees)	15.9 ± 3.4	15.1 ± 3.3	*p* = 0.62
F_L_ (cm)	7.8 ± 1.6	8.6 ± 2.8	*p* = 0.51

Analysis via unpaired t-test

*Abbreviations: CSA* Cross-sectional area, *M*_*T*_ Muscle thickness, *P*_*A*_ Pennation angle, *F*_*L*_ Fascicle length

### Mitochondrial function

#### Extrinsic function

**Fig. 2:**
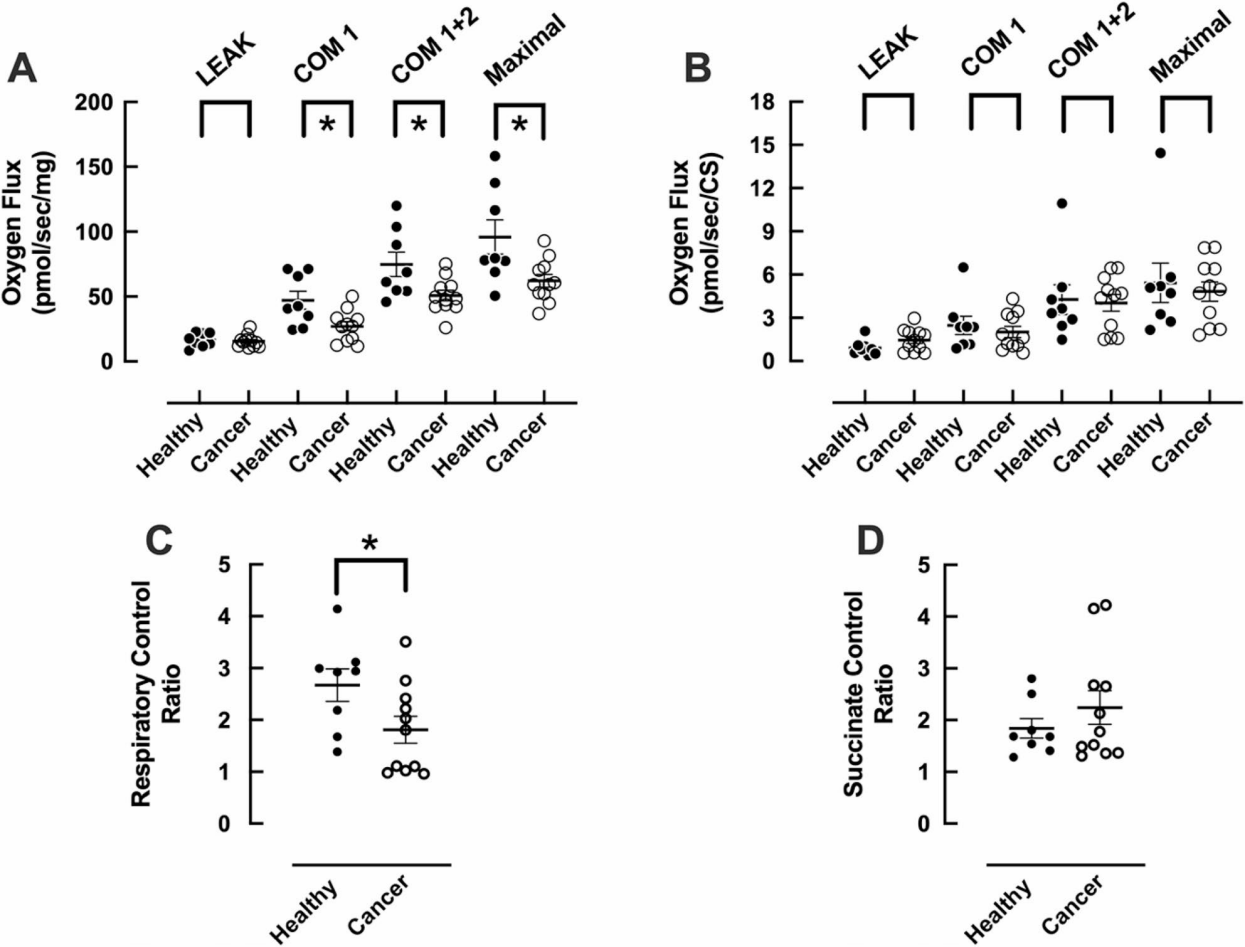
Respiratory characteristics of permeablised muscle fibres. Extrinsic (**A**) and intrinsic (**B**) mitochondrial oxidative phosphorylation and mitochondrial control (respiratory control ratio (RCR) (**C**) and succinate control ratio (SCR) (**D**)) in prostate cancer patients (*n* = 12) and healthy individuals (*n* = 8) matched for sex (male), age and body mass index. Data are mean ± SEM. Analysis via parametric unpaired t-test: *=*p* < 0.05

Assessing extrinsic OXPHOS function (OXPHOS relative to sample weight), there was no significant difference in LEAK between the groups (healthy: 16.6 ± 5.3 vs. cancer: 15.6 ± 4.7, *p* = 0.67) (Fig. 2A). There were however significant differences in maximal complex I activity (healthy: 47.1 ± 19.7 vs. cancer: 27.1 ± 12.1, *p* = 0.01) (Fig. 2A), maximal complex I + II activity (healthy: 74.8 ± 26.7 vs. cancer: 50.7 ± 13.4, *p* = 0.01) (Fig. 2A) and maximal activity (healthy: 95.9 ± 37.4 vs. cancer: 62.1 ± 16.3, *p* = 0.02) (Fig. 2A), with values for each of these parameters lower in the cancer cohort compared to healthy controls.

#### CS activity

There was no significant difference in CS activity between the two groups (healthy: 22.2 ± 12.1 vs. cancer: 14.0 ± 6.0, *p* = 0.08).

#### Intrinsic function

Assessing intrinsic OXPHOS function (OXPHOS normalised to CS activity), there was no significant difference between the healthy controls and cancer patients at any stage (Fig. 2B; Table 2).

**Table 2:** Parameters of skeletal muscle intrinsic mitochondrial oxidative phosphorylation in prostate cancer patients (*n* = 12) and healthy individuals (*n* = 8) matched for sex (male), age and body mass index

	Healthy controls	Cancer patients	*P*-value
LEAK (pmol/sec/CS)	0.9 ± 0.5	1.5 ± 0.8	*p*=0.11
Complex I (pmol/sec/CS)	2.5 ± 1.8	2.0 ± 1.3	*p* = 0.52
Complex I + II (pmol/sec/CS)	4.3 ± 2.9	4.0 ± 1.9	*p* = 0.84
Maximal (pmol/sec/CS)	5.4 ± 3.8	4.8 ± 2.2	*p* = 0.67

Analysis via unpaired t-test

#### Mitochondrial control

In permeablised muscle fibres, RCR was significantly lower in the cancer cohort when compared to the healthy cohort (healthy: 2.7 ± 0.9 vs. cancer: 1.8 ± 0.9, *p* = 0.04) (Fig. 2C). There was no significant difference in SCR between the two cohorts (healthy: 1.8 ± 0.5 vs. cancer: 2.2 ± 1.1, *p* = 0.35) (Fig. 2D).

### RT-PCR

Mitochondrial fission, fusion and CS expression was significantly higher in cancer patients compared to healthy controls. PGC-1α expression was also significantly higher in cancer patients. There was no significant difference in COX-II expression between the two groups (Table 3).

**Table 3:** Reverse transcription polymerase chain reaction (RT-PCR) data in prostate cancer patients (*n* = 12) and healthy individuals (*n* = 8) matched for sex (male), age and body mass index

	Healthy controls	Cancer patients	*P*-value
Mitochondrial Fission			
DNM1L	1.2 ± 0.8	4.5 ± 2.1	0.0005
FIS1	1.0 ± 0.3	1.4 ± 0.4	0.04
Mitochondrial Fusion			
MFN1	1.2 ± 0.7	2.3 ± 0.8	0.003
MFN2	1.1 ± 0.5	1.9 ± 0.5	0.005
OPA1	1.2 ± 0.7	2.9 ± 0.9	0.0002
Other			
Citrate synthase	1.1 ± 0.4	1.7 ± 0.4	0.003
PGC1-α	1.2 ± 0.9	2.1 ± 0.7	0.02
COX-II	1.2 ± 0.7	0.8 ± 0.3	0.17

Analysis via unpaired t-test

*Abbreviations: DNM1L* Dynamin-related protein 1, *FIS1* Fission protein 1, *MFN* Mitofusin, *OPA1* Optic Atrophy 1, *PGC-1α* Peroxisome proliferator-activated receptor gamma coactivator 1-alpha, *COX-II* Cyclooxygenase-2

## Discussion

Comparing early-stage prostate cancer patients to healthy volunteers matched for sex, age and BMI, functional performance indicators including handgrip strength, TUG time, and SPPB scores were not different between the groups. Similarly, SKM architecture of the VL, including CSA and M_T_; both of which have been shown to represent muscle size, were not different between the groups. Despite this concordance in measures representing muscle mass and physical function- two of the key features of cancer cachexia [33, 34], a discernible difference in SKM OXPHOS, specifically extrinsic function, was apparent between prostate cancer patients and healthy controls.

The lower extrinsic SKM OXPHOS seen in the pre-cachectic prostate cancer patients is manifested by a decline in maximal flux and complex I linked stimulated respiration. This difference in extrinsic function, especially when coupled with the similar levels of intrinsic mitochondrial function, echoes findings from, to our knowledge, the sole other study assessing SKM OXPHOS function in human cancer patients [22]. It also aligns with the findings of Neyroud et al., who observed lower maximal mitochondrial respiration rates in tumour bearing compared to healthy mice [21]. However, this study utilises CS activity as a marker of mitochondrial content which is not a direct marker of mitochondrial content, meaning its interpretation maybe limited. In addition, the SUIT protocol utilised focuses on NADH and succinate linked respiration, although these are two key components of carbohydrate respiration, this protocol does not assess other respiration metabolites.

This pattern of reduced extrinsic mitochondrial function but preservation of intrinsic function implies a change in SKM mitochondrial content or remodelling altering area, not intrinsic dysfunction, a suggestion supported by pre-clinical studies. In a mouse model, White et al., demonstrated a 2.5-fold increase in the fission marker FIS1 and declines in the fusion markers Mfn1 and Mfn2, resulting in smaller mitochondria [35]. Other pre-clinical studies have reported cancer to be associated enlarged, distorted, or disrupted mitochondria, along with reduced mitochondrial mass [36–38]. Within our study, we found an increase in fission markers but curiously also found increased fusion markers, suggesting higher mitochondrial turnover. Work within breast cancer patients has also shown changes in metabolic pathways [39], which could contribute to the extrinsic decline in SKM OXPHOS with preservation of intrinsic mitochondrial OXPHOS – this is supported within our cohorts by the lower expression of PGC1-α.

The second difference in SKM OXPHOS function in the prostate cancer cohort is a disturbance in RCR, which is indicative of mitochondria’s ability to transition from a resting to an active state [40]. Interestingly, Kunz et al., observed a higher RCR in their human cancer cohort compared to healthy controls [22], a finding at odds with our data reported herein and those from animal models of cancer where RCR is reported to be impaired [20, 21–41]. Our finding of RCR being ~ 30% lower in cancer patients than in healthy controls aligns with the data of Brown et al., who reported RCR to be 25% lower in a rodent model of cachexia [20]. Impaired RCR is likely to contribute to increased muscle fatigue due to an inability for energy supply to match demand, which may drive cachexia directly and/or indirectly via reduced physical activity levels due to such fatigue.

It was likely our exclusion criteria which meant that our participants with cancer had no measurable evidence of cachexia (i.e., all presented with preserved muscle mass and function), and future studies should aim to include participants who display features of cachexia to better understand the relationship between SKM mitochondrial function and this condition. Further, exploring this relationship in different cancer types may provide insight into the differing impact of cancer type on SKM physiology. This study is limited by a failure to assess participants physical activity levels, something that is strongly linked to mitochondrial function [42], and which future studies need to include. Finally, a larger study would provide further confidence in the results reported herein.

## Conclusions

In conclusion, there was lower SKM extrinsic OXPHOS in prostate cancer patients, in the absence of muscle mass or function losses, when compared to age-matched disease-free volunteers. This appears to be due to changes in mitochondrial content or remodelling, with preservation of intrinsic function. This was seen in a cancer cohort with organ confined disease, who were scheduled for a planned curative resection without neoadjuvant chemotherapy. Prior publications have previously demonstrated lower OXPHOS in in mixed-origin metastatic cases, all of whom were receiving palliative chemotherapy [22], and whom as such, represent a markedly different population in terms of cancer burden. This key finding herein supports the notion driven by pre-clinical evidence, that alterations in SKM function are potentially an early impact of malignancy and as such a potential driver of cancer cachexia [20, 21], which warrants further exploration.

Future research should aim to fully determine the mechanisms underpinning malignancy-induced changes in SKM OXPHOS in humans. Elucidating these mechanisms will likely advance the development of targeted strategies to mitigate the development and impact of cancer cachexia, and as such overall well-being in individuals with cancer.

## Electronic Supplementary Material

Below is the link to the electronic supplementary material.

Supplementary Material 1: PCR primer design details.

## Data Availability

The datasets analysed during the current study are available from the corresponding author upon reasonable request.

## Abbreviations

SKM: Skeletal muscle
OXPHOS: Mitochondrial oxidative phosphorylation
BMI: Body mass index
TUG: Timed up-and-go
SPPB: Short physical performance battery
CSA: Cross-sectional area
M_T_: Muscle thickness
P_A_: Pennation angle
F_L_: Fascicle length
RT-PCR: Real-time polymerase chain reaction
CS: Citrate synthase
DNM1L: Dynamin-related protein 1
FIS1: Fission 1 protein
MFN1: Mitofusin 1
MFN2: Mitofusin 2
OPA1: Optic atropy 1
COX-II: Cyclooxygenase-2
PGC1-a: Peroxisome proliferator-activated receptor gamma coactivator 1-alpha
SUIT: Substrate uncoupled inhibitor titration
TCA: Tricarboxylic acid cycle
RCR: Respiratory control ratio
SCR: Succinate control ratio
IQR: Interquartile range
COM: Complex
