# Supplementary material for: A cohort study investigating differences in skeletal muscle mitochondrial function between prostate cancer patients and healthy controls
Source: BMC Cancer. 2026 May 21;26:965. doi: 10.1186/s12885-026-16164-2 (PMC13465824; doi:10.1186/s12885-026-16164-2)
Supplement: Supplementary file 1 [file 12885_2026_16164_MOESM1_ESM.docx]

## PCR primer design

Primers were designed using the Primer-BLAST (https://www.ncbi.nlm.nih.gov/tools/primer-blast/) primer designing tool, and primer validation was achieved with efficiency determination through standard curves and melting curve analysis. The following primers were used in this study:

| **Gene** | **Forward** | **Reverse** |
| --- | --- | --- |
| **MFN2** | CACCACCAATTGCTTCCTGC | AGCTGGTTCACAGTCTTGGC |
| **OPA1** | CTGGCGGAAGACCTCAAGAA | AGGCTGGACAAAAGACGTTGA |
| **DNM1L** | AGGAGAAGAAAATGGGGTGGA | ATTGGTTCAGGGCTTACTCC |
| **FIS1** | GGTGCGGAGCAAGTACAATG | GCCTTCTCGTATTCCTTGAGC |
| **MFN1** | GCTGGACCACCCATGCCAAG | CTTGTTGGCACAGGCGAGCA |
| **Citrate synthase** | CCTGCCTAATGACCCCATGTT | CATAATACTGGAGCAGCACCCC |
| **PGC1-α** | GACCCTCCTCACACCAAACCCA | ACCCTTGGGGTCATTTGGTGACT |
| **COX-II** | TCACCTTTCATGATCACGCCC | AGGACTAGGATGATGGCGGG |
